# Lupane-type triterpenes and their anti-cancer activities against most common malignant tumors: A review

**DOI:** 10.17179/excli2016-642

**Published:** 2016-11-30

**Authors:** MH Cháirez-Ramírez, MR Moreno-Jiménez, RF González-Laredo, JA Gallegos-Infante, Nuria Elizabeth Rocha-Guzmán

**Affiliations:** 1 Instituto Tecnológico de Durango, Departamento de Ingenierías Química y Bioquímica, Blvd. Felipe Pescador 1830 Ote., Col Nueva Vizcaya, 34080 Durango, Dgo., México

**Keywords:** lupeol, betulin, betulinic acid, cancer, signaling pathway

## Abstract

In recent times, a great deal of interest has been motivated on plant derived compounds known as nutraceuticals. These compounds exert important beneficial activities that improve people's health status when are consumed regularly, and now they appear as a viable option to explore their possible therapeutic effects against diseases like cancer. Particularly, lupane-type triterpenes have shown great ability to modulate multiple cancer-related signaling pathways and processes, including NF-κB, Wnt/β-catenin, PI3K/Akt, apoptosis, and many other routes related to proliferation or cell death, which are uncontrolled in malignant tumors. These investigations have promoted *in vitro* and *in vivo* studies, searching their mechanisms of action; although more research is still needed to prove its potential in human clinical trials. This review focuses on the ability of betulin, betulinic acid and lupeol to show benefits against the most common types of malignant tumors, which are considered a major global threat for public health.

## Introduction

In present times, non-communicable chronic diseases are responsible for about 63 % of deaths worldwide. This group includes diabetes mellitus (DM), cardiovascular diseases (CVD), chronic respiratory diseases (CRD) and cancer, being responsible for approximately 38 million of deaths per year; 75 % of these deaths (28 million) occur commonly in third world countries.

Cancer is a generic term that comprises a large number of diseases that affect distinct parts of the human body. It is characterised by uncontrolled cell growth, and is capable to disseminate to different tissues from where it was originated (metastasis), leading to people´s death. Cancer is responsible for one in every seven deaths worldwide (14.1 million of cancer's new cases are detected that causes 8.2 millions of deaths per year), causing even more deaths than the acquired immunodeficiency syndrome (AIDS), tuberculosis and malaria combined, making it a serious public health problem. Malignant tumors are the second leading cause of death in high-income countries (5.3 million of deceases) after CVD, and third cause in middle-low income countries (2.9 million of deaths) after CVD and parasitic-infectious diseases (American Cancer Society, 2015[2]). Principal cancer types that caused more deaths in 2012 were lung (1.59 millions), liver (745,000), gastric (723,000), colorectal (694,000), and breast cancer (521,000) (Stewart and Wild, 2014[77]).

Cancer's chemoprevention has become increasingly important in modern times, and implies the use of natural, synthetic or biological agents to reverse, suppress or prevent early stages of carcinogenesis, as well as the progression of pre-malignant cells to an invasive disease (Sporn, 1976[76]). Even though there are many treatments to different cancer types (i.e. surgery, chemotherapy, radiation therapy, targeted therapy and immunotherapy), these treatments have shown many collateral effects on patients. A balanced cancer inhibition involves prevention, early diagnosis, effective treatment, and includes palliative therapy. 

Nowadays, it is known that plants produce a wide range of phytochemicals (non-nutritional compounds, i.e. secondary metabolites) and some of such compounds are credited as health promoters. It is also important to note that people are more concerned on taking care of their diet, having a higher intake of plant-derived products. These products might contain phytochemicals that could exert biological activities against chronic-degenerative diseases such as cancer, thus contributing to improve health status of people. These biological activities are explained by the presence of nutraceuticals. This term was used the first time in 1989 by Dr. Stephen DeFelice combining the terms “Nutrition” and “Pharmaceutical”. Most of nutraceuticals are safer, less toxic and with less side effects compared to synthetic drugs (McAlindon, 2006[56]). Some nutraceuticals show chemopreventive activities, inhibiting or interfering with the process of disease (Surh, 2003[78]); among nutraceuticals phytosterols seem to be a promising group of bioactive compounds. 

Phytosterols include plant sterols and stanols. They are natural occurring compounds that can be found in many plants derived foods and food supplements, mainly those rich in oil content or products derived therefrom. Average intake of these compounds in normal diets ranges between 178 to 463 mg per day (Andersson et al., 2004[5]). 

Terpenes are members of the phytosterol family, and can be found in a wide variety of living organisms like prokaryotic cells as well in eukaryotic organisms (plants and animals). However, composition of plants has shown to be richer on these compounds with biological activity than any other organisms. These compounds are derived from isoprene units (C_5_H_8_), and are classified according to their number of carbons, thus they are named such as mono-(C10), sesqui-(C15), di-(C20), sester-(C25), tri-(C30) and tetra-(C40) terpenes. Isoprene units can be acyclic or polycyclic (mono, bi, tri, tetra or penta). According to their complexity these compounds can be found in different parts of plants, simple terpenes (mono or sesquiterpenes) are found in essential oils of plant raw materials, while more complex terpenes (i.e., triterpenes) are found in resins and balms (Muffler et al., 2011[61]). 

**Figure 1:**
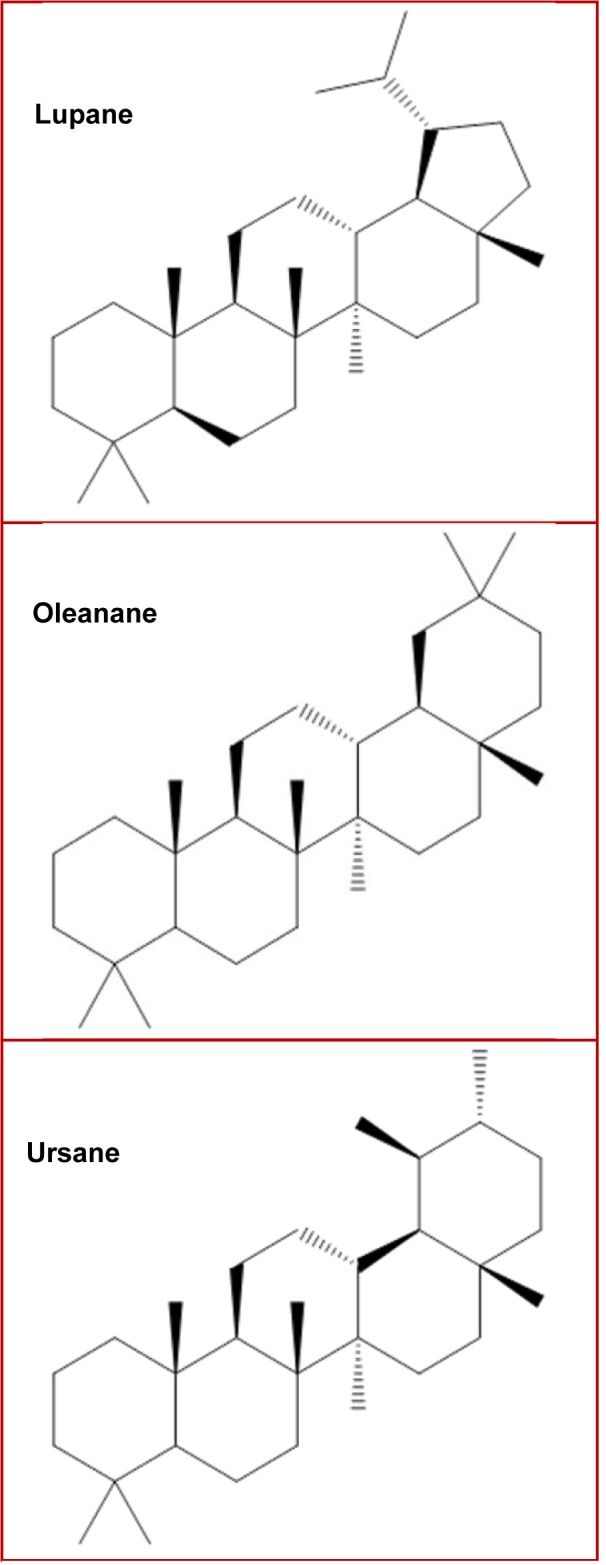
Principal pentacyclic triterpenes structures.

A group of terpenes with particular importance are triterpenes; about 30,000 different triterpenes have been identified and classified according to their structures and chemical properties, and can be found in the form of aglycones or as free acids. The most common triterpene structures include pentacyclic (taraxanes, oleananes, ursanes, lupanes and baccharanes) and tetracyclic (dammarane, curcubitane and euphanes), being oleananes and ursanes the most abundant in higher plants (Dzubak et al., 2006[16]; Nazaruk and Borzym-Kluczyk, 2015[62]; Yin, 2015[95]). Emphasizing from biological perspective, the most important triterpenes are the pentacyclic oleanane, ursane, lupane (Figure 1(Fig. 1)), and the tetracyclic dammarane, euphane structures (Muffler et al., 2011[61]). 

**Figure 2:**
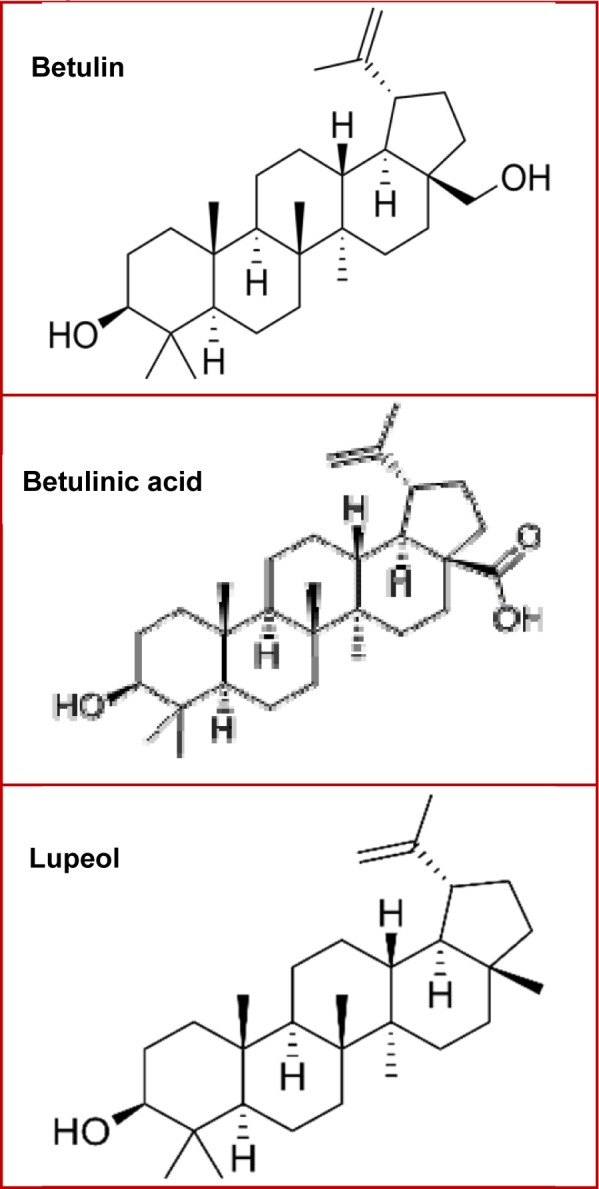
Figure 2: Chemical structures of principal lupane-type triterpenes

Triterpenes are often recognized as bioactive compounds, and are subjected to multiple phytochemical and pharmacological studies. Several research studies have been conducted in recent times in order to elucidate the mechanisms and modulatory activity in different signaling pathways, by which these compounds exhibit benefits against different cancer types. This review focuses on natural triterpenes especially the lupane-type triterpenes betulin, betulinic acid and lupeol (Figure 2(Fig. 2)), and their activities against the most common cancer types among men and women, highlighting the benefits of these compounds in prevention and treatment of malignant tumors.

## Signaling Pathways Involved in Cancer

Cancer is characterized by an abnormal cell growth due to aberrant gene expression patterns. Most of cancers are acquired by alteration of genetic codes (caused by the accumulation of mutations in somatic cells). Normal cells have very strict regulatory mechanisms between cell growth, division and apoptosis, maintaining a homeostatic state. Cancer cells show independence from this growth signals, due to specific mutations on particular genes that control proteins involved on intracellular signaling pathways. Growth signals independence shown by cancer cells allow them to enter on the cell cycle regardless of external stimuli (Foster, 2008[24]). The enormous genomic instability within cancer cells, leads to immortality and fast cell and tissue transformation (Artandi and DePinho, 2009[7]). Generally, principal genes affected in cancer can be divided into two groups: oncogenes and tumor suppressor genes. Oncogenes (which are phenotypically dominant, *i.e*., only one mutation in an allele is enough to cancer promotion) regularly suffer gain function mutations, like gene amplification (overexpression) or production of mutant proteins with unusual activity, favoring growth, division and enhanced survival of cancer cells. On the other hand, tumor suppressor genes (which are phenotypically recessive, *i.e*., it is necessary a function loss of the two alleles towards cancer promotion) have the function to inhibit cell proliferation and survival. Tumor suppressor genes are often related to control cell cycle progression and apoptosis, because these genes are responsible for preventing uncontrolled cell growth, DNA repair, and also are responsible for regulation of multiple cell cycle control checkpoints (Doucas and Berry, 2006[13]; Weinstein et al., 2008[88]; Zhang et al., 2007[96]).

Some examples of proteins (oncogene mutation) most commonly mutated in cancer are: non-receptor tyrosine kinases (nRTKs; *e.g*. Src), receptor tyrosine kinases (RTK; *e.g*. EGFR, ErbB), serine/threonine kinases (*e.g*. Akt), transcription factors (*e.g*. NF-κB, Myc), small GTPases (*e.g*. Ras), lipid kinases (*e.g*. PI3K) and nuclear receptors (*e.g*. ER), cell cycle regulators (e.g. cyclins), and proliferative pathways (*e.g*. Wnt). Deletions lead to inactivation of tumor suppression proteins, such as PTEN (antagonist of the PI3K/Akt pathway), retinoblastoma protein (pRb), cyclin-dependent kinase inhibitors (CKI), p53, etc. Protein p53 is probably the most studied in cancer research and is known as the guardian of genome. Protein p53 is activated when some damage is detected on DNA, triggering apoptotic signals and stopping cell cycle progression, hence it has high relevance in studies of anticancer activities (Lee and Muller, 2010[42]; Sever and Brugge, 2015[73]; Weinberg, 2013[87]).

## Anti-Cancer Activities of Lupane-Type Triterpenes

### Lung cancer

Lung cancer possesses the highest prevalence and mortality worldwide, its life expectancy for over five years is fateful (only 16 %); even with chemotherapy less than 20 % of patients live more than five years (Keith and Miller, 2013[40]). Combined with these dismal numbers, advances in lung cancer treatment still remain poor compared to other types of malignant tumors such as breast, prostate and colon, because surgery results unfeasible. In this sense, many researches have been conducted in order to determine whether lupane-type triterpenes exhibit some benefit on this type of malignant tumor. Cancer is consequence of many genetic changes where normal cells become malignant, being the disruption of programed cell death one of the main mechanisms of cancer (Hanahan and Weinberg, 2000[31]). Apoptosis has been linked long time ago with the elimination of cancer cells and tumor progression (Kerr et al., 1972[41]). Betulin has shown to induce apoptosis on lung cell line A549, when apoptosis induction was increased by 27 % compared with untreated control, showing an IC_50 _(20 µM) almost equal to cisplatin (25 µM) (Pyo et al., 2009[68]). Cisplatin is well known as an apoptosis inductor (Barry et al., 1990[8]), however betulin has not toxic effects even at high doses (Makarova et al., 2011[54]), which is an advantage compared to cisplatin, because the latter causes nephrotoxicity and is the main restriction for its dosing (Miller et al., 2010[59]). Similar results for apoptosis induction using betulin were found by Rzeski et al. (2009[70]) against the A549 cell line. Apoptotic effects of betulin could be promoted by the apoptosis intrinsic pathway (Li et al., 2010[46]). Some other activities against lung cancer have been shown by betulin. Betulin is capable of increasing expression levels of p21 and p27, and reducing levels of Cyclin B, Cyclin D1, and Cyclin E, thus it inhibits cell proliferation through regulation of these checkpoint proteins of the cell cycle. Inhibition of cell cycle progression is also another important mechanism on cancer research. Cell cycle is a highly-regulated process by the activation of Cdk/cyclin complexes. The p21 and p27 proteins are family members of Cip/Kip and their principal function is the inhibition of kinase activity of Cdk/cyclin complexes in G_1_/S and G_2_/M phases, then regulation of both proteins is altered in cancer (Mitrea et al., 2012[60]). AMPK is also activated by betulin with a decreased phosphorylation of mTOR and S6 kinase (Li et al., 2014[45]). AMPK is a highly-conserved pathway; in situations when nutrients are scare, AMPK regulates cell growth mainly by suppressing the mTORC1 pathway (by direct phosphorylation of tumor suppressor TSC2 or RAPTOR) (Mihaylova and Shaw, 2011[57]). Betulinic acid presents cytotoxic effects against A549 and H1650 lung cancer cell lines, because it shows IC_50_ values of 8.92±1.68 and 7.25±1.54 µg/mL, respectively. Betulinic acid shows also *in vivo* activities on tumor reduction and increased expression of p38, p-JNK and Bax, and reduction of the anti-apoptotic marker Bcl-2. Expression levels of pro-apoptotic markers such as cleaved caspase-3 and cleaved caspase-8 were significantly increased (Godugu et al., 2014[26]), so this activity from betulinic acid can be beneficial, because cancer cells usually do not exhibit apoptosis as do normal cells. Betulinic acid shows effects against lung cancer growth by regulation of specificity protein 1 (Sp1) and triggering apoptosis (Hsu et al., 2012[36]). Transcription factor Sp1 regulates genes involved in cell proliferation like cyclin D1, c-Jun, and c-Myc (Wierstra, 2008[89]). Another study suggests that growth inhibition of malignant lung tumors by betulinic acid are through Sp1 degradation (Hsu et al., 2015[35]). Pentacyclic triterpene lupeol has also been reported with anti-cancer activities against lung cancer. Lupeol has shown downregulation on COX-2 on A549 cell line (Sankaranarayanan et al., 2013[72]). COX-2 is a well-known marker of cancer; its overexpression has been found in lung tumors, being related to a poor prognosis. COX-2 participates in different steps of cancer like increasing cell proliferation, affecting apoptosis, and thus influencing anti-cancer drugs effectiveness (Sobolewski et al., 2010[74]). The 3β-O-succinyl-lupeol (LD9-4), a derivative from lupeol, has shown to induce autophagy via downregulation of the mTOR pathway and upregulation of Beclin 1 (Hao et al., 2011[32]). Autophagy is an important homeostatic cell process that controls the catabolism of proteins and organelles, which are processed in autophagosomes, digested in lysosomes, and recycled to maintain cellular homeostasis. Autophagy acts as a tumor suppressor, which is its role in cancer, by preventing the accumulation of damaged proteins and cell organelles (Yang et al., 2011[93]). 

### Hepatic cancer

Hepatic cancer has two principal types: intrahepatic bile duct cancer (ICC) and hepatocellular carcinoma (HCC). Worldwide liver cancer possesses high incidence; hepatocellular carcinoma is by itself the sixth most common cancer and is the third leading cause of cancer-related deaths (Forner et al., 2012[23]). Liver cancer is twice common in men than in women, and its five-years survival rate ranges 10-20 %, being one of the poorest prognoses among cancers (American Cancer Society, 2015[2]). The HCC is the most common liver cancer type, and is associated in many cases with the hepatitis virus or cirrhosis. HCC is a disease with important regional differences, and its causes may vary significantly by region. In Eastern Asia and sub-Saharan Africa countries, about 80 % of the incidence of HCC is related to hepatitis B virus (HBV) as well as exposure to aflatoxin B1. Otherwise, in Europe, North America and Japan, the main risk factors are high alcohol consumption (~40-60 g/day increases the risk about 1.5-2.0 times to develop HCC), and the hepatitis C virus (HCV) (~15-20 times prone to develop the disease) (Donato et al., 2002[12]; El-Serag, 2011[18]; Forner et al., 2012[23]). 

Some biological activities of lupane-type triterpenes have been investigated. Results of these scientific investigations found that they are able to show benefits against risk factors that trigger liver cancer. Lupeol has hepatic-protective activities against aflatoxin-B1 induced liver damage, restoring activity levels of hepatic enzymes LDH, AST, ALT, ALP and reducing oxidative stress trough glutathione and antioxidant enzymes, normalizing their levels, and counterbalancing depletion of the antioxidant systems (Preetha et al., 2006[67]). Oxidative stress is an imbalance in reactive oxygen species (ROS) production, and the capacity of endogenous antioxidant system (SOD, CAT, and glutathione system) to control its concentration in the body, less than 5 % are toxic to the body if they increase their concentration in the organism (Sosa et al., 2013[75]). If ROS are produced by prolonged time period, they can alter biomolecules such as proteins, lipids and DNA. These DNA alterations can be turned into mutations leading to genomic instability, cell proliferation and possible neoplastic transformation (Fang et al., 2009[19]; Visconti and Grieco, 2009[85]). Ethanol consumption is also anther risk factor for hepatic cancer development. Ethanol produces liver fibrosis; in this sense betulin and betulinic acid are capable of downregulating CYP2E1 activity, this enzyme plays a crucial role on ethanol-induced fatty liver and in alcohol detoxification. Alcohol toxicity is decreased when CYP2E1 is inhibited (Lu and Cederbaum, 2008[52]). Betulinic acid is also capable of preventing hepatic alcoholic damage by improving the antioxidant system (increasing the activity of SOD, CAT, GPx and reducing the levels of malondialdehyde in mice treated with betulinic acid) (Yi et al., 2014[94]). Alcohol-induced liver damage with ethanol was attenuated by betulin and betulinic acid by downregulating ROS production, preventing NF-κB and JNK phosphorylations, and playing a crucial role on inflammation prevention (Szuster-Ciesielska et al., 2011[79]). Betulinic acid also exhibits beneficial activities against HCV by downregulation of COX-2 expression at transcriptional level by preventing NF-κB phosphorylation and later DNA binding (Lin et al., 2015[48]). Nowadays, it is well known that high abnormal activities of inflammatory transcription factors (*i.e*. NF-κB, JNK) and cytokines can be related to cancer development (Mantovani et al., 2008[55]). In this sense, lupane-type triterpenes offer remarkable beneficial effects on liver cancer-related risk factors. 

The observed beneficial effects of lupane-type triterpenes on hepatic cancer highlights the great potential of this type of compounds for the prevention and possible treatment of this disease. Anti-cancer activity of triterpenes extracts (with principal components such as betulin, lupeol and betulinic acid) against HCC cells derived from a xenograft model was achieved due to an increase in caspase 3/7. This extract had less cytotoxic activity on healthy hepatocytes, and required eight times more concentration to cause some disturbance on cell viability. This anti-hepatocellular carcinoma activity from triterpene extracts was through the intrinsic apoptosis pathway (Hertrampf et al., 2012[33]). Undoubtedly, triterpenes show the advantage of non-toxic effects on healthy cells and being selective against cancer cells (Zuco et al., 2002[97]). Betulinic acid is capable of apoptosis induction in hepatoblastoma cells through PI3K/Akt growth inhibition and caspase 3 activation (Eichenmüller et al., 2009[17]). Lupeol suppresses tumorigenesis in HCC cells by reducing CD133 and modulation of PTEN-Akt pathway (Lee et al., 2010[43]). The potential against liver cancer of betulinic acid could be related to its antitumor activity and apoptosis induction by p53-regulated p66^shc ^(Yang et al., 2015[91]). The PI3K/Akt pathway is one of the main cell survival pathways. It is activated by different types of cellular stimuli (but also activated by toxic agents) and regulates fundamental cellular functions such as proliferation, growth, transcription, cell cycle and apoptosis (Cantley, 2002[9]; Vanhaesebroeck and Alessi, 2000[84]). All these regulatory activities shown by lupane-type triterpenes give them a promising future in research and development of new therapeutic agents for the treatment of liver cancer.

### Gastric cancer

Gastric cancer is the fifth most common cancer and remains as one of the leading cancer-related deaths around the world (Ferlay et al., 2014[20]). Stomach cancer is only exceeded by lung and liver cancers in death incidences. Gastric cancer is quite common in countries like Japan, China, Korea, Russia and regions such as Central and South America (Torre et al., 2015[82]). Principal stomach cancer type is adenocarcinoma with about 90 % of the incidences, other gastric malignant tumors types occur as lymphomas and sarcomas, etc. (Karimi et al., 2014[39]). Many risk factors have been associated to gastric cancer development like smoking tobacco, dietary patterns, poor vegetables and fruits intake, uncontrolled consumption of non-steroidal anti-inflammatory drugs (NSAIDs), obesity and *Helicobacter* *pylori* chronic infection with about 75-89 % of cases in this type of cancer (Plummer et al., 2014[66]). Gastric cancer still has poor prognosis, its five- year life expectancy rate is about 20 % (Karimi et al., 2014[39]).

Typical gastric cancer treatment includes surgery, which is undoubtedly the only potentially curative treatment for stomach cancer. An increase on patients' survival rates has been observed by combining treatments such as chemotherapy or radiotherapy with surgery (Orditura et al., 2014[63]). However, chemotherapeutical drugs always carry side effects. In this sense, research with lupane-type triterpenoids has found interesting biological activities against this pathology, without the undesirable side effects caused by chemotherapy or could help to enhance the effects of the chemotherapeutical drugs. Betulin dinicotinate has shown protective effects against indomethacin and ethanol gastric induced-ulcers (Flekhter et al., 2002[21]). Lupeol also possesses protective properties on ethanol-induced gastric damage (Lira et al., 2009[49]). It is well known that ulcers can be related to stomach cancer development, thus betulin and lupeol could act as good preventive agents. Betulin and related compounds have shown interesting protective activities. Betulinic acid is capable to exhibit a better cytotoxic effect than betulin against human gastric drug-resistant cell lines 257P, 257RNOV, and 257RDB, being the IC_50_ for betulin in the range of 10.97-18.74 µM and for betulinic acid 2.01-6.16 µM (Drag et al., 2009[14]). Cytotoxic effects from betulin and betulinic acid extracted from *Belamcanda chinensis* against human gastric cancer cell line MGC-803 have been reported (Liu et al., 2012[50]). Betulin can induce intrinsic apoptosis pathway on SGC7901 human gastric cancer cell line through caspase 3/9 upregulation, and intracellular ROS played a crucial role in this apoptosis induction process (Li et al., 2016[47]). Betulinic acid induces apoptosis on human gastric adenocarcinoma (AGS) cell line, through cell cycle arrest on G2/M phase; cyclin B2 expression was down-regulated by the betulinic acid treatment (Yang et al., 2010[92]). Cell cycle regulation is always important for cancer treatment, mainly because cancer cells are incapable for regulating its own growth. Cyclin B1/CDK1 complex has a crucial role in cell cycle progression; it is the checkpoint to G2/M phase transition and subsequent onset of mitosis (Pines and Hunter, 1990[64]). The ability of lupane-type triterpenes in apoptosis and cell cycle regulation is highly important in the quest for new therapeutic agents for treatment of cancer. Recent approaches have found that lupeol possesses anti-gastric cancer activities. Lupeol is capable of enhancing the cytotoxic function of natural killer (NK) cells against gastric cancer cells (Wu et al., 2013[90]). This result is very important due to NK cells playing an important role in tumor development, and many models have shown that deficiency on these immune cells causes more aggressive tumors (Waldhauer and Steinle, 2008[86]). 

Furthermore, a recent research suggests that lupane-type triterpene lupeol displays a chemo-sensitization and enhancement of the inhibitory effect shown by 5-fluorouracil (5-FU) in gastric cancer, inducing apoptosis by upregulation of Bax (pro-apoptotic protein) and p53, and downregulation of survivin and Bcl-2 (anti-apoptotic protein) (Liu et al., 2016[51]). All these results taken together suggest a great anti-gastric cancer potential by such compounds, and could act as potential candidates for stomach cancer treatments, always with the awareness that lupane-type triterpenes are not substitutes to current treatments.

### Colorectal cancer

Colorectal cancer (CRC) is the third most common malignant tumor type (1.4 million new cases per year are reported), and is the fourth leading cause of cancer-related deaths worldwide, causing approximately 694,000 (~8 % of total cancer-related deaths) mortalities annually (Ferlay et al., 2014[20]). This disease is related to age, and is principally diagnosed on mature adults (~50 years old), while in young people it is quite rare. Average age for diagnosis is around 66 years for males and 70 years for females (Miller et al., 2016[58]). Colorectal cancer has important geographical differences, because it is more common in developed countries with a western lifestyle like Europe, North America, and Australia; and lower risk incidence is found in countries of Africa, South America and Asia (Center et al., 2009[10]; Haggar and Boushey, 2009[30]). Colorectal cancer unlike the other cancer types commented above in this review does not have a dismal prognosis; its five-year survival rate is about 65 % and the rate for 10-year survival is 58 % (Miller et al., 2016[58]), these are encouraging numbers for such a serious condition as this cancer is recognized. Principal risk factors associated to colorectal cancer development includes age and inflammatory bowel disease (IBD); these risk factors increase 4-20 times the possibility for colorectal cancer development more than in healthy people (Janout and Kollarova, 2001[37]), although family history, genetic factors, nutritional habits, alcohol consumption, obesity and smoking are also important risk factors (Haggar and Boushey, 2009[30]). Like in any other cancer type, CRC treatment has different procedures: surgery, chemotherapy (5-FU, oxaliplatin), radiotherapy and targeted therapies (bevacizumab, cetuximab), being surgery treatment the most used (in early stages 0 and I, surgery may be used without any other type of complementary therapy) (American Cancer Society, 2014[3]; Hagan et al., 2013[29]). It is remarkable, that lupane-type triterpenes have shown interesting effects against CRC, which will be mentioned below and their multiple effects makes them promising in the treatment of this disease. Betulinic acid has been capable of preventing the NF-κB activation and its nucleus translocation, preventing cell proliferation and activating extrinsic apoptosis pathway (TNF-α dependent apoptosis) (Takada and Aggarwal*,* 2003[80]). Betulinic acid also showed specific apoptosis induction by caspase 3/7 upregulation, and cytotoxic activity against colon cancer cell line (HCT 116), with a minimum cytotoxic effect on normal colonic cells (CCD-18Co) (Aisha et al., 2012[1]). The preference shown by lupane-type triterpenes for cancer lines, makes them ideal for cancer treatment, because they only exhibit their effects on malignant cells. Anti-CRC effects of betulinic acid have also been proven *in vivo*. Betulinic acid inhibited tumor growth possibly by downregulating VEGF (vascular endothelial growth factor) expression at transcriptional level in a xenograft model (Ren et al., 2010[69]). VEGF is known to regulate angiogenesis and vascular permeability; angiogenesis is an important process in tumor growth and development (Goel and Mercurio, 2013[27]), because new blood vessels feed the tumor with oxygen and nutrients, allowing tumor development. Betulinic acid has interesting effects on drug-resistant CRC cells, compared to other standard drugs in CRC therapy such as 5-FU, IRT, and OXT; its mechanism of action suggests that betulinic acid triggers apoptosis since the levels of cleaved caspase 3 are increased (Jung et al., 2007[38]). Autophagy is another process that is induced by an analogue of betulinic acid on HT-29 cells by increasing the level of Beclin 1 (autophagy-related protein) and decreasing levels of p62 (Dutta et al., 2016[15]). Cell autophagy is a process in which stressed cells undergo to lysosomal degradation of defective organelles and damaged proteins (Levine and Klionsky, 2004[44]). Under high stress conditions, apoptosis deficient malignant cells may die by other mechanisms, improving the efficiency of some therapies (Gözüaçık and Kimchi, 2007[28]; Yang et al., 2011[93]). Lupeol also possesses anti-CRC potential, because modulates the Wnt/β-catenin signaling pathway a hallmark from this pathology, this potential is displayed through inhibition of Wnt/β-catenin transcriptional activity and decreasing the level of β-catenin in nuclei (Tarapore et al., 2013[81]). Wnt/β-catenin pathway is one of the main critical control points for cell proliferation and cell polarity, thus promoting cell homeostasis (MacDonald et al., 2009[53]). All these activities support the important biological potential of lupane-type triterpenes in CRC management, and open the possibility that people take more awareness on plants naturally occurring compounds as potential therapeutic agents. 

### Breast cancer

Breast cancer is by now the second most common malignant tumor worldwide, the leading cause of cancer-related deaths in females, and the fifth of all cancer cases. This pathology had about 1.67 million of new cases (about 25 % of all cancers reported) and caused about 522,000 deaths in 2012. Breast cancer has the highest incidence rates in Western Europe and United States, while the lowest incidences occur in Asia and Africa (Ferlay et al., 2014[20]; Torre et al., 2015[82]). Principal associated risk factors include being female, aging, genetic predisposition (BRCA1 and BRCA2 mutations), breast density, radiation exposure, nulliparity, long term hormonal therapy, and life style associated factors (obesity, sedentarism, etc.) (American Cancer Society, 2015[4]). The survival rate to this pathology has increased considerably in recent years, mostly due to prevention campaigns, self-exploration, and the development of more effective therapies. Survival rate has increased from 75 % in the 70's to 90 % in the first decade of 2000 (Saadatmand et al., 2015[71]). The five-year life expectancy is about 82.1 % (Arrington et al., 2014[6]). Breast cancer treatment is based on type of molecular markers expressed, such as estrogen receptor (ER), progesterone receptor (PR) and HER2/neu. Principal treatments for this disease include surgery, radiotherapy chemotherapy, and hormonal therapy (American Cancer Society, 2015[4]). Lupeol, betulin and betulinic acid have shown good results against breast cancer in many conducted researches. Methanolic extract from *Nardostachys jatamansi *(lupeol rich plant) were tested against ER^+^ cell line MCF-7 and ER^-^ MDA-231, being more effective against MDA-231, promoting cell cycle arrest on G2/M phase and apoptosis induction (Chaudhary et al., 2015[11]). This result is relevant due to ER^+^ breast cancer tumors are hormone responsive, indicating that lupeol could exert effects on ER^-^ tumors such as basal or triple negative, which are the most difficult mammary tumors to treat, because are unresponsive to typical hormone therapy. Another study demonstrated that lupeol isolated from *Elephantopus scaber*, exerts its apoptotic activity through Bcl-2 and Bcl-xL downregulation, leading to cytochrome c release and intrinsic apoptosis pathway induction. This effect was only seen on MCF-7 (breast cancer), but not so on MCF-10A (normal cell line) (Pitchai et al., 2014[65]). However, it is possible that the effect of lupeol against breast cancer is limited as recently demonstrated, and that lupeol had no capability against invasive cells (Fu et al., 2015[25]). Betulin and betulinic acid are capable of selective apoptosis induction on invasive breast cancer cells, but not so in normal breast cells by upregulation of Bax, cleaved caspase-3, and cleaved PARP, increasing p53 and p21 levels and decreasing p-Akt (Hsu et al., 2015[34]). Betulinic acid-rich fraction from *Dillenia suffruticosa* roots led to upregulation of p53, p21, and increasing the protein ratio of Bax to Blc-2 by 2, resulting on apoptosis induction (Foo et al., 2015[22]). Betulinic acid also showed important effects on growth inhibition of ER^-^ cells, which is a relevant finding since breast cancer ER^-^ related tumors do have limited endocrine-based therapies (Tzenov et al., 2015[83]). 

## Conclusions

Interest for plant derived natural products as therapeutic agents has recently increased worldwide. Particularly, lupane-type triterpenes such as betulin, betulinic acid and lupeol have shown multiple bioactivities against different cancer cell lines and hold encouraging antitumor effects. Their activity might be promising against lung, liver, stomach, colorectal and breast cancers. Further research *in vitro and in vivo*, and human clinical trials are required to elucidate their mechanisms of action and their whole therapeutic potential. However, it exists great expectation for this class of compounds as therapeutic agents for combating malignant tumors.

## Conflict of interest

Authors state no conflict of interest

## Acknowledgements

Author M.H.C.R. acknowledges graduate scholarship from CONACYT (National Council of Science & Technology) and also financial support for a basic research project CONACYT-SEP-CIENCIA BASICA (grant number 241241).
